# The Influence of the Different Disposition Characteristics of Snake Toxins on the Pharmacokinetics of Snake Venom

**DOI:** 10.3390/toxins12030188

**Published:** 2020-03-16

**Authors:** Suchaya Sanhajariya, Geoffrey K. Isbister, Stephen B. Duffull

**Affiliations:** 1 Clinical Toxicology Research Group, University of Newcastle, Newcastle 2298, Australia; geoff.isbister@gmail.com; 2 School of Pharmacy, University of Otago, Dunedin 9016, New Zealand; stephen.duffull@otago.ac.nz

**Keywords:** snakes, venom, pharmacokinetics, stochastic simulation and estimation

## Abstract

Snake venom is comprised of a combination of different proteins and peptides with a wide range of molecular weights and different disposition processes inherent to each compound. This causes venom to have a complex exposure profile. Our study investigates 1) how each molecular weight fraction (toxin) of venom contributes to the overall time course of the snake venom, and 2) the ability to determine toxin profiles based on the profile of the overall venom only. We undertook an *in silico* simulation and modelling study. Sixteen variations of venom, comprising of two to nine toxins with different molecular weights were investigated. The pharmacokinetic parameters (i.e., clearance, CL, and volume of distribution, V) of each toxin were generated based on a log-linear relationship with molecular weight. The concentration–time data of each toxin were simulated for 100 virtual patients using MATLAB and the total concentration–time data of each toxin were modelled using NONMEM. We found that the data of sixteen mixtures were best described by either two- or three-compartment models, despite the venom being made up of more than three different toxins. This suggests that it is generally not possible to determine individual toxin profiles based on measurements of total venom concentrations only.

## 1. Introduction

Snake envenomation is defined as the injection of snake venom into human (or animal) tissue through specialised teeth or “fangs”. Snake venom contains a complex mixture of enzymatic and non-enzymatic proteins (toxins), which cause various biological effects. Many toxins are clinically important and contribute to the significant number of deaths and disabilities reported worldwide, particularly in resource-poor countries [1]. An understanding of the time course, denoted pharmacokinetics (PK), of snake venom in humans is crucial for improving snake envenomation management strategies. In particular, an understanding of the exposure profile will help determine how long snake venom remains in the system and therefore the likely benefits of administering antivenom after various periods post-bite.

In the past three decades, a number of studies had been conducted to investigate the PK of snake venom in animals (e.g., [2,3,4,5,6,7,8,9,10,11,12,13]) and following human envenomation (e.g., [14,15,16,17]). A review of the pharmacokinetic analyses of these data indicated that the most common disposition model used was a two-compartment model, although one- and three-compartment models were also reported [18]. It is important to note that snake venoms are made up of a combination of different proteins, which have molecular weights ranging from 4 to 150 kDa. Compounds (in this case toxins) of different molecular weights are expected to exhibit different PK characteristics. The overall venom PK profile is therefore governed by the combination of disposition processes inherent to each compound. The PK parameters obtained from whole venom studies will be an averaged representation over the individual profiles of the contributing toxins. Such studies may not capture the underlying characteristics of individual venom toxins and the variability of the various snake toxin disposition processes in the biological system. It is possible, in some circumstances, that the profile of venom may be dominated by a few highly abundant toxins, which could then be identified from the overall venom profile. Since different molecular weight proteins in snake venom can cause different biological effects [18], an understanding of their PK characteristics may provide an insight into the time course of toxic effects and the relevance of different timed administrations of polyclonal antivenom. We postulate that it might be possible, with prior knowledge of the expected toxin composition and abundance in a venom, to identify potential toxins from the whole venom profile.

In this study, we aim to determine 1) the effects of different molecular weight proteins/toxins on the overall disposition profile of snake venom, and 2) the ability to extract different toxin profiles, given the whole venom profile.

## 2. Results

One hundred virtual datasets were created for 100 virtual patients. Each patient provided 12 blood samples equivalent to timed venom concentrations. Each dataset was constructed by summing timed venom concentration data taken from the simulated PK profiles of the individual component toxins. These virtual timed venom concentration profiles were then modelled. Akaike information criterion (AIC) values of successfully minimised models were assessed to determine the best-fit model.

### 2.1. Intraveneous Bolus Venom Administration (“Best Field Case” Scenario)

**Figure 1:**
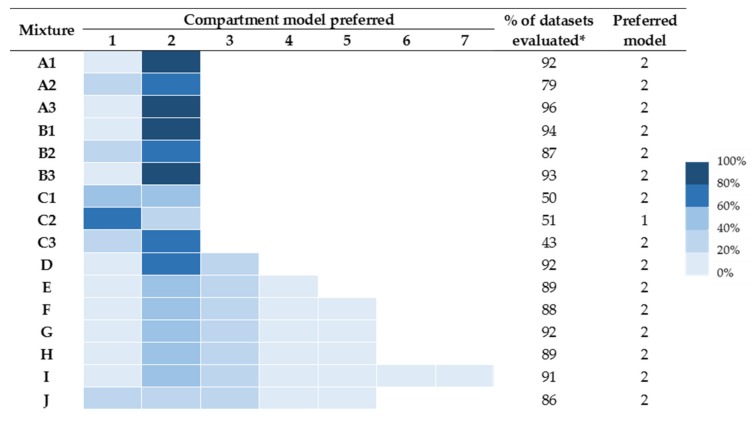
Fitting results of models under the “best field case” scenario. Coloured chart represents the percentage of times the particular compartmental model was preferred. (*) denotes the % of successful runs.

A summary of the preferred models is provided in Figure 1. In no circumstance was more than a seven-compartment model preferred, and hence support for the eight- and nine-compartment models is not available (and not shown). The number of successful runs varied from 43% to 96%. In general, runs provided good convergence properties except mixtures for C (mixtures of two toxins with similar molecular weights).

The concentration–time data of all mixtures evaluated were best described by two-compartment models, except for mixture C2, which was best described by a one-compartment model. Four- and six-compartment model fits were preferred for some mixtures containing more than four components. For mixture J (representative of *Hypnale hypnale*), it can be seen that the number of runs that preferred one-, two-, and three-compartment models was similar.

### 2.2. The Depot Compartment Model (“Likely Field Case” Scenario)

**Figure 2:**
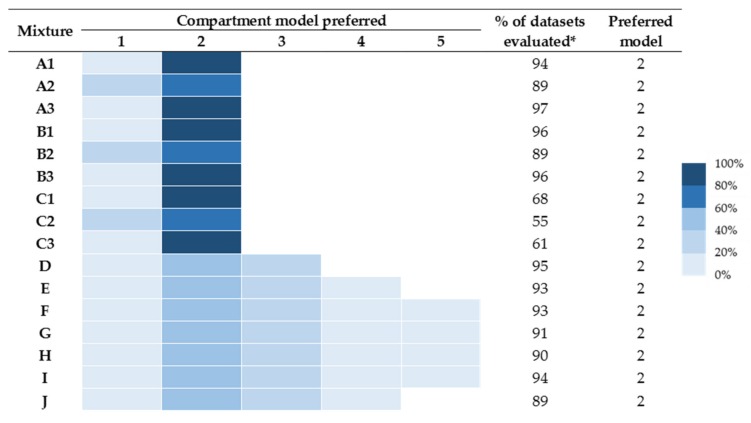
Fitting results of models under the “likely field case” scenario. Coloured chart represents the percentage of times the particular compartmental model was preferred. (*) denotes the % of successful runs.

A summary of the preferred models is provided in Figure 2. In no circumstance was more than a five-compartment model preferred, and hence data for the six- to nine-compartment models are not shown. The number of successful runs varied from 55% to 96%. Again, runs provided good convergence properties, except mixtures for C (mixtures of two toxins with similar molecular weights).

In this scenario, data of all sixteen mixtures were best described by two-compartment models. The three-compartment model was next preferred for mixtures D–J, with less preference for one- and four-compartment models.

### 2.3. Distinguishing Individual Toxin Half-Lives from the Whole Venom

**Figure 3:**
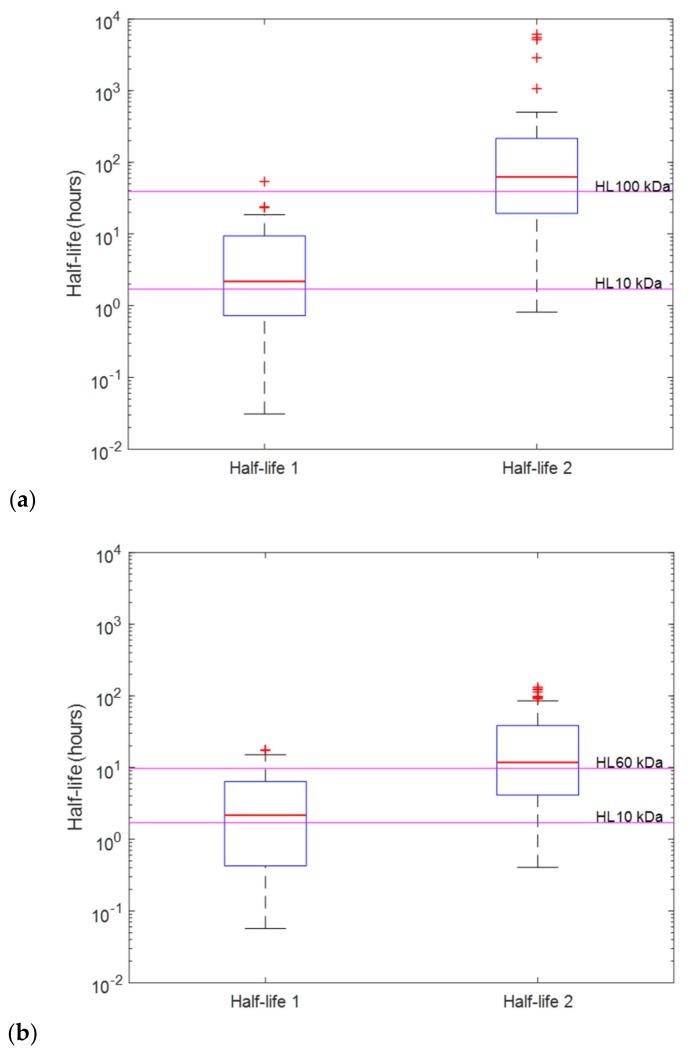
Half-lives of (**a**) mixture A1 (comprised of 1:1 ratio of 10 and 100 kDa toxin fractions) and (**b**) mixture B1 (comprised of 1:1 ratio of 10 and 60 kDa toxin fractions) calculated from two-compartment pharmacokinetic (PK) parameters of each run in the “likely field case” scenario. On each box, the central red line indicates the median, and the bottom and top edges of the box indicate the 25th and 75th percentiles, respectively. Magenta lines are the nominal half-lives (calculated from the clearance, *CL*, and volume of distribution, *V*, of the prior) of toxins based on their molecular weights.

**Figure 4:**
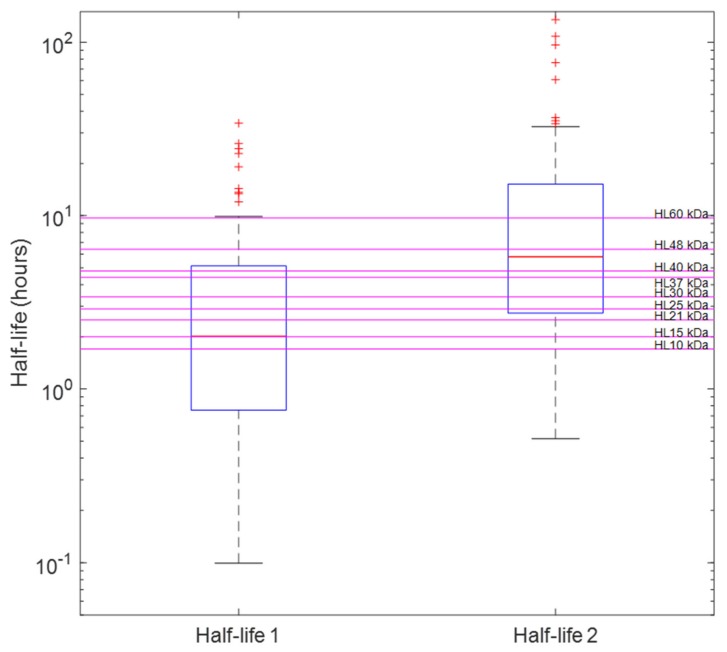
Half-lives (distribution and elimination) of mixture J (*Hypnale hypnale*) calculated from two-compartment PK parameters of each run in “the likely field case” scenario. On each box, the central red line indicates the median, and the bottom and top edges of the box indicate the 25th and 75th percentiles, respectively. Magenta lines are the nominal half-lives (calculated from the *CL* and *V* of the prior) of toxin based on their molecular weights (10,15, 21, 25, 30, 37, 40, 48, and 60 kDa).

Half-lives calculated from PK parameters obtained from successfully minimised models were compared to the nominal half-lives of the venom composites. Box plots were used to visually examine whether the whole venom profile can be used to determine the PK profiles of individual toxins. Figure 3a,b demonstrates examples (mixture A1 and B1) of the cases where it may be possible to determine the half-lives of the dominating toxins from the whole venom. Here the mean half-lives of the two-compartment disposition kinetics are relatively similar to half-lives of the venom composites. On the other hand, data from venoms containing three or more toxin fractions did not support the identification of more than two- or three-compartment disposition kinetics, so no more than three half-lives can be identified. Figure 4 illustrates an example (mixture J) where we cannot determine all of the half-lives of individual toxin fractions. Here, we were only able to identify two compartmental half-lives from the mixture, which do not represent (but do encompass) all half-lives of toxins in mixture J (*H. hypnale*).

## 3. Discussion

This study provides a starting point for improving our understanding of the time course of snake venom/toxin exposure after envenomation. A population-based simulation and estimation approach was utilised to investigate how the PK of the composites/toxins in snake venoms can affect the whole venom PK profile, and whether or not we can identify the individual toxin profiles from the whole venom PK profile. The data generated in this study account for the variability between toxin composition of different snake venoms and differences between individuals and how they handle individual toxins. The use of the prior for the population values for *CL* and *V* allows us to account for the variability in the characteristics of different molecular weight proteins. Although the mixtures were defined *a priori* in this simulation (in Section 5.1.2), the variability in the *CL* and *V* parameters for the whole venom from the prior can also be considered as variability in the toxin profile between snakes.

The concentration–time data of sixteen different admixtures of virtual toxins were investigated in this study. They were all shown to exhibit two- or three-compartmental disposition behaviours in all three scenarios evaluated: the perfect case (Figure S1.1 of the Supplementary Materials), the best field case, and the likely field case. This was despite many of the mixtures comprising more than three toxins with widely different molecular weights and PK characteristics. This finding is consistent with previous PK studies in which no more than three-compartment PK behaviour was reported following an intravenous or intramuscular administration of snake venom in animals [2,3,4,5,6,7,8,9,10,11,12,13], and following uncontrolled observational studies of snakebite in human patients [15,16,18]. Our simulated “*H. hypnale* venom” data was best described by a two-compartment model (with a slightly lower preference for a three-compartment model), similar to the animal (rabbit) study, in which *H. hypnale* venom was shown to exhibit three-compartment behaviour [10]. 

It is also interesting to note that only in the “perfect scenario” (Figure S1.1 of the Supplementary Materials) a preference for a three-compartment model was seen in mixtures containing three or more toxins. Whereas in the scenarios in which uncertainty was incorporated in the simulated population PK in the prior (“best field case” and “likely field case”), the venom data were mostly described by two-compartment disposition kinetics. This is in line with our previous review which reported that snake venoms commonly exhibit two-compartmental behaviour in animal studies [18]. This phenomenon suggests that the variability attached to *CL* and *V* of each different molecular weight toxin leads to the overlapping of toxin PK characteristics. Thus, making it difficult to distinguish individual toxin profiles from that of the whole venom. This will subsequently lead to an underestimation of the true number of compartments in the PK model for snake venom and will affect the ability to identify the half-life of individual toxins of interest. 

Identifying the dominant toxin profile from the PK characteristics of a multi-compartment venom profile can be useful for determining the time course of important toxins in relation to their biological effect. However, this study demonstrates the difficulties in determining individual toxin profiles based on their half-lives from the venom admixture when there are more than three dominant toxins present, which is usually the case for snake venoms. Because of this, venom PK alone does not provide a complete picture of the different toxin PK and we should not over-interpret PK studies when explaining the time course of biological effects in association with particular toxin fractions. However, it does seem that the range of half-lives evident from the toxins do encompass the half-lives of the preferred two-compartment model. If specific information is required on the exposure to a specific toxin, it will therefore be necessary to develop a monoclonal assay of appropriate sensitivity and specificity for different toxins.

Although a stochastic simulation and estimation approach is a valuable tool to virtually investigate the disposition processes of snake venom and its composites, there are some limitations that may not truly reflect the clinical scenarios. Firstly, molecular weight (MW) was used to predict the pharmacokinetics of the toxins in the body following envenomation. While molecular size is shown to affect the clearance and the volume of distribution of proteins/toxins (as demonstrated in Appendix A and Section S2 (Table S2.1, Table S2.2, and Figures S2.1–S2.4) of the Supplementary Materials), we acknowledge that there are other factors that may affect the PK characteristics of toxins, such as immunogenicity and presence of endogenous proteases and peptidases. Secondly, the absorption rate constant of each venom component was assumed to be the same and was set to 1.4 h^−1^. Different sized molecular weight toxins are likely to have different absorption processes, but (in relation to the disposition profile) are unlikely to be different enough to affect interpretation. In addition, we have not accounted for fang length (long-fanged viperid versus short-fanged elapid), which may play an important determinant in whether the venom is injected subcutaneously or intramuscularly. Hence, there may be a depot effect with large volume and deep injection of venoms from large vipers such as Russell’s viper (*Daboia russelii).* We acknowledge, having an intensive sampling scheme (i.e., 12 samples per patient) and samples available as early as 0.25 h does not occur commonly in the clinical setting, as patients may arrive at the hospital anywhere from half an hour to more than 6 h after being bitten [14,19,20,21], so typically real data collected tend to be fewer and more sparse than was the case in this study. This may explain why snake venom often exhibits only one-compartment behaviour in PK studies in humans [15,16,18]. Whereas in the situation in which the whole profile can be studied, such as in this study or animal models, a multi-compartment behaviour is observed [2,3,4,5,6,7,8,9,10,11,12,13]. Finally, it is possible that some venoms may themselves display multi-compartment disposition kinetics, which would further muddy the ability to disentangle toxic compositions from whole venom.

## 4. Conclusions

We utilised a stochastic simulation and estimation approach to evaluate the effect of the pharmacokinetics of different snake venom composites on the overall disposition characteristics of snake venom observed in animal or clinical studies. A population pharmacokinetic analysis of venom data does not support the identification of more than a three-compartment profile. This indicates that it is not feasible to differentiate the individual toxin profile of venoms based on the data of whole venom only, with exception to circumstances where there are a small number of highly expressed determinants.

## 5. Materials and Methods

Both aims of this study were addressed using a stochastic simulation estimation (SSE) study using MATLAB version 2018a (The MathWorks, Inc., Natick, MA, USA) for simulation and NONMEM version 7.3 (ICON Development Solutions, Ellicott City, MD, USA) for population PK modelling and estimation using first-order conditional estimation method with interaction.

In this SSE study a venom ‘dose’ was constructed from a set of characteristic proteins and then administered to each virtual subject. We use the term characteristic to denote that these are proteins that have similar molecular weights that are characteristic of typical toxins. Each simulated venom consisted of a discrete set of toxins, each with a different mixture of molecular weights. The study consisted of 100 virtual patients who provided an intensive sampling protocol of 12 blood samples for total venom concentration (the sum of all toxins). Note this study is designed to evaluate the best-case scenario, and we do not anticipate that such a study would necessarily be practical in the clinical/field setting. The resultant timed venom concentrations were then analysed using compartmental pharmacokinetic models in NONMEM.

### 5.1. Simulation of the Venom Profile

The venom profile used in this SSE study was based on the Sri Lankan hump-nosed pit viper (*H. hypnale*) venom. Tan et al. [22] have identified a typical venom profile (protein components, molecular weights, and relative abundances). The summary of their finding is shown in Table 1. It is noted that venom composition may vary within species based on region and between species. Hence, this example is indicative only.

The pharmacokinetic characteristics of the typical venom constituent toxins of *H. hypnale* are not available in the literature, and, therefore, each of their profiles was based on the typical characteristics associated with similar (therapeutic) molecular weight proteins. These proteins formed the basis of the prior pharmacokinetic information that was used for simulating different toxin profiles.

#### 5.1.1. Establishing Prior PK Parameters for Characteristic Proteins

**Figure A1:**
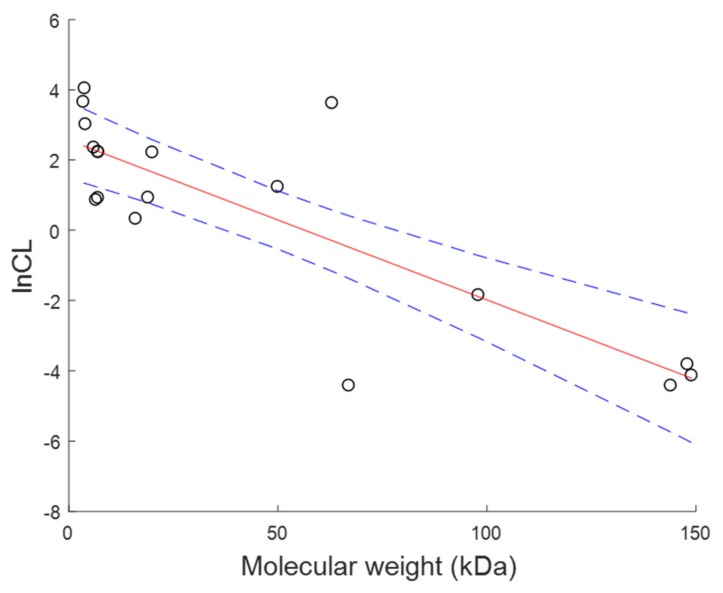
Plot of clearance versus molecular weight of molecules. Red line is the least squares regression line and blue dash lines are 95% confidence interval. Regression equation: lnCL=−0.04548×MW+2.566, uncertainty ($\sigma_{\mathrm{prior}}$): 1.6528.

**Figure A2:**
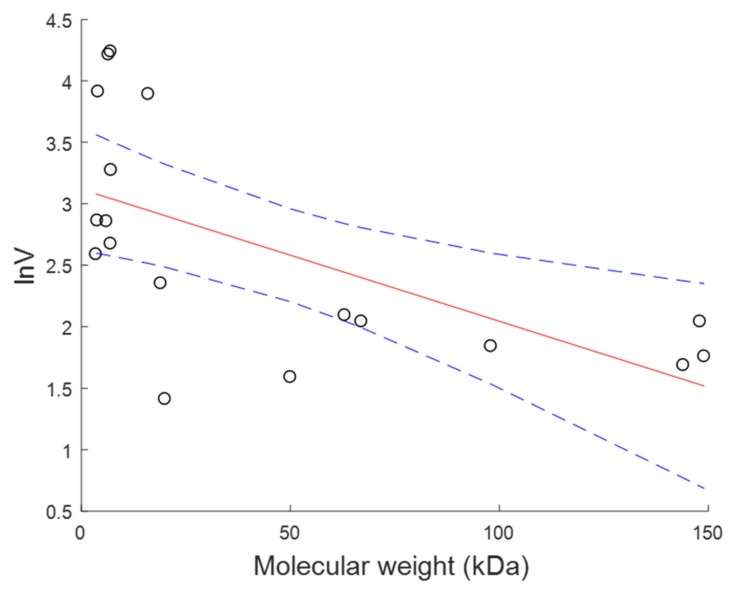
Plot of volume of distribution versus molecular weight of molecules. Red line is the least squares regression line and blue dash lines are 95% confidence interval. Regression equation: lnV=−0.01073×MW+3.117, uncertainty ($\sigma_{\mathrm{prior}}$): 0.7525.

A non-exhaustive list of protein drugs and their relative molecular weights and PK parameters was created. The values of the parameters clearance (*CL*) and volume of distribution (*V*) were collated (Table A1 of the Appendix A). The population PK parameters (*CL* and *V*) were regressed against the molecular weight and a log-linear model was found to provide the best fit to the data (Figure A1 and Figure A2 of Appendix A) with additive residual error representing the uncertainty in the prior.

#### 5.1.2. The Mixtures

Sixteen different toxin mixtures comprising two to nine toxin components (Table 2) were investigated. Each toxin mixture represents a virtual venom. Mixture J, in this simulation, contains similar molecular weight fractions to the dominant proteins present in *H. hypnale* venom. The total venom dose ($D_{tot})$ in these simulations was the sum of the different “doses” of the *n*-toxin components ($D_{1}$, …, $D_{n}$), given by:

(1) $$D_{tot}=D_{1}+D_{2}+\ldots +D_{n}.$$

Since the dose of venom is not known, the total dose was indexed to 10,000 where $\sum_{i=1}^{n}D_{i}=10,000$ and the component toxins were considered as proportions of the total amount (note the value of 10,000 is arbitrary and any value could be used). A number of simple venom compositions were considered initially (A1, …, F) to determine their influence on the overall venom profile. Mixtures G–I represent more realistic and complicated venom compositions and mixture J, the most realistic venom composition.

#### 5.1.3. The Simulation

Concentration–time data of each toxin in the sixteen different mixtures (A1–J) were generated with a rich sampling scheme for 100 virtual patients. The venom concentration, the sum of toxin concentration–time data, will be used as the dependent variable for the subsequent PK modelling process.

The simulation was based on a hierarchical model. Firstly, the population parameters for each toxin were simulated from the prior, followed by the individual parameters for each toxin based on the population parameters mean and variance parameters. Finally, the concentration–time data were simulated for each individual (based on the individual’s parameter values for each toxin) with residual error. The equations and table with parameter values used in the simulation are provided in Appendix B.

#### Model for Population Parameters for Each Toxin Based on Molecular Weight

The mean population PK parameters values ($\bar{CL}$ and $\bar{V}$) of each toxin were generated based on their molecular weight from the regression equation and associated residual error identified in the prior (Section 5.1.1). Simulation of the population parameters from the prior took the form, shown for *CL*:

(2) $$\ln\left({\bar{\theta }}_{CL}\right)\sim N\left(g\left(\beta ,\;\mathrm{kDa}\right),{\;\sigma }_{\mathrm{prior},\mathrm{lnCL}}^{2}\right),$$

where $\bar{\theta }$ is the typical value for the population, N is a univariate normal distribution, $g\left(\beta ,\;\mathrm{kDa}\right)$ is the regression model (Appendix A) with parameters β, and $\sigma_{\mathrm{prior}}^{2}$ is the prior uncertainty associated with the model.

#### Model for the Individual Parameters for Each Toxin

Individual values of *CL* and *V* were simulated from the population values assuming an exponential between subject variability model. The model used for simulating individual parameters took the form, shown for *CL*:

(3) $$\ln\left(\theta_{CL_{i}}\right)\sim N\left(\ln\left({\bar{\theta }}_{CL}\right),\;\omega_{CL}^{2}\right),$$

where $\theta_{i}$ is the individual value of the parameter, N is a univariate normal distribution, $\bar{\theta }$ is the population parameter value with a between subject variance of $\omega_{\theta }^{2}$, reflecting between-subject variability (BSV). BSV in this study is assumed to be 44% for *CL* and 30% for *V* (as reported in [18]).

#### Model for the Concentration–Time Data of Toxins

Two structural models were considered for the data: (1) an instantaneous intravenous (IV) input model (equivalent to a direct injection of the venom into a capillary) and (2) a depot absorption model (equivalent to injection of the venom into subcutaneous tissue with subsequent absorption into the circulation). These models are provided in Equations (4) and (5), respectively. Model 1 is anticipated to be a simplification of the input process, which would provide the cleanest disposition venom profile. It was included here to investigate the best-case scenario.

(4) $$C_{n}\left(t\right)=\frac{D_{n}}{V_{n}}\times e^{-\left(\frac{CL_{n}}{V_{n}}\right)\times t},$$

where $C_{n}\left(t\right)$ is plasma concentration of the *n*^th^-venom component as a function of time t, $D_{n}$ is dose administered, $V_{n}$ is apparent volume of distribution, and $CL_{n}$ is apparent clearance.

(5) $$C_{n}\left(t\right)=\frac{D_{n}\times F\times k_{a}}{V_{n}\times \left(k_{a}-\frac{CL_{n}}{V_{n}}\right)}\times \left(e^{-\left(\frac{CL_{n}}{V_{n}}\right)\times t}-e^{-k_{a}\times t}\right),$$

where F is bioavailability (in this study, it is fixed to 1), and $k_{a}$ is absorption rate constant (in this study, $k_{a}$ is fixed to 1.4 h^−1^, which gives rise to an absorption half-life of 0.5 h and a time to peak concentration of approximately 1.5 h).

Concentration–time data of each toxin in the mixture were generated for 12 sampling time points over a duration of approximately four half-lives of the toxin with longest half-life in the venom mixture. The sampling times (in hours) were 0.5, 1, 2, 4, 12, 24, 48, 72, 96, 120, 144, and 168 for mixture A1, A2, and A3; 0.25, 0.5, 0.75, 1, 1.5, 2, 2.5, 3, 4, 6, 10, and 14 for mixture C1, C2, and C3; and 0.25, 0.5, 1, 1.5, 2, 2.5, 4, 8, 12, 24, 30, and 40 for mixture B1, B2, B3, D, E, F, G, H, I, and J, with the earliest sampling time being 0.25 h post-envenomation.

The venom concentration was the sum over each of the n toxins (separately for the IV bolus model and the tissue depot compartment models).

A combined error model for residual unexplained variability of population data was incorporated into the venom concentration–time data (Equation (6)). This is to describe the magnitude of uncertainty or the unexplained differences between the observations and the predictions from the model.

(6) $$y\left(t\right)\sim N\left(f\left(\theta_{i},x\right),\;\Sigma \right),\;\mathrm{with}\;\Sigma =\sigma_{prop}^{2}\cdot \;f{\left(\theta_{i},x\right)}^{2}+\sigma_{add}^{2},$$

where y is the observed venom concentration (the sum of the toxin concentration at a given time (t)), N is a univariate normal distribution, $\theta_{i}$ is a vector of individual parameter estimates for the *i*^th^ individual, and x are the independent variables (e.g., dose and time), with error matrix Σ, reflecting residual unexplained variability (RUV). Proportional error $\sigma_{prop}$ and additive error $\sigma_{add}$ were assumed to be 0.217 and approximately 0.001 (as per [18]). The residual error variances were set to be the same for both the instantaneous IV input model and the depot absorption model. Finally, the function $f\left(\theta_{i},x\right)$ represents a compartmental model, which is allowed to range from 1- to *n*-compartments and the best structure is based on statistical criteria.

This simulation consisted of 16 venoms each administered either by instantaneous IV injection (IV bolus) or into a depot compartment with each patient providing 12 samples for a total of 100 patients per study and the study replicated 100 times; representing a total of 320,000 simulated patients.

### 5.2. Pharmacokinetic Modelling

The venom data were modelled under two scenarios:

i) An IV bolus considering using a 1- to n- compartment model (best field case).
ii) A first-order absorption using a 1- to n- compartment model (likely field case).

The PK parameters of the compartment models were estimated for each of a one-compartment model and extended up to a nine-compartment model as the data allowed (the total number of toxins present in venom mixture J). A reduction in AIC values of models that were reported to have minimised successfully was used as a basis for model selection. Smaller AIC values represent models that are statistically preferred. Models that failed to minimise were recorded but were not used to inform the best compartmental model. Unsuccessful minimisation scenarios were not investigated further.

An additional scenario was considered where, an IV bolus one-compartment model using the population parameter estimates without uncertainty in the prior, i.e., setting the variances of the prior σ for both *CL* and *V* to zero. This represents the perfect case scenario and is useful only for comparator purposes. These results are provided in a Supplementary file.

## Figures and Tables

**Table 1:** Relative venom components of the Sri Lankan hump-nosed pit viper (*H. hypnale*) (modified from Tan et al. (2015) [22]).

Protein Components	Approximate Molecular Weight (kDa)	Relative Abundance
Phospholipase A_2_ (PLA_2_)	14–15	40.1%
Zinc-dependent snake venom metalloprotease (SVMP)	25	29.8%
	30	6.4%
	40	0.7%
L-amino acid (LAAO)	60	11.9%
C-type lectin (CTL)	14	5.5%
Snake venom serine protease (SVSP)—Thrombin-like enzyme ancrod	37	0.5%
Snake venom serine protease (SVSP)—Thrombin-like enzyme	48	2.8%
Not determined	9–10	0.8%
	15	0.7%
	21	0.8%

**Table 2:** Dose fraction (in percentage) of toxin components in mixtures investigated in this study.

Mixture	Molecular Weights (kDa)									
	10	15	21	25	30	37	40	48	60	100
A1	50	.	.	.	.	.	.	.	.	50
A2	20	.	.	.	.	.	.	.	.	80
A3	80	.	.	.	.	.	.	.	.	20
B1	50	.	.	.	.	.	.	.	50	.
B2	20	.	.	.	.	.	.	.	80	.
B3	80	.	.	.	.	.	.	.	20	.
C1	50	.	.	.	50	.	.	.	.	.
C2	20	.	.	.	80	.	.	.	.	.
C3	80	.	.	.	20	.	.	.	.	.
D	.	34	.	33	.	.	.	.	33	.
E	.	25	.	25	25	.	.	.	25	.
F	.	20	.	20	20	.	.	20	20	.
G	17	17	.	17	17	.	.	16	16	.
H	14	14	14	14	14	.	.	15	15	.
I	12.5	12.5	12.5	12.5	12.5	.	12.5	12.5	12.5	.
J	0.8	46.3	0.8	29.8	6.4	0.5	0.7	2.8	11.9	.

## Supplementary Materials

The following are available online at https://www.mdpi.com/2072-6651/12/3/188/s1, Figure S1.1: Fitting results of models under the “perfect case” scenario. Tables S2.1 and S2.2, Figures S2.1 and S2.2: Further examples that highlight this association between pharmacokinetic parameters and molecular weight. Figures S2.3 and S2.4: Comparison of relationship between pharmacokinetic parameters and molecular weight between the two examples and our current study.

## Appendix A

**Table A1:** Reported human clearance and volume of distribution for protein drugs and allometrically scaled human parameter values from animal studies of snake venom toxins.

Compounds	Molecular Weight (kDa)	*CL* (L·h^−1^)	*V* (L)	References
Nesiritide	3.5	38.6^a^	13.3^a^	#
Glucagon recombinant	3.8	56.7^a^	17.5^a^	#
Abaloparatide	4.0	20.4^b^	50.0	#
Relaxin	6.0	10.5	17.4	[23]
Neurotoxin (*Naja sumatrana*)	6.5	2.36^†^	67.6^†c^	[11]
Lepirudin	7.0	9.30*	14.5*	#
Cardiotoxin (*N. sumatrana*)	7.0	2.50^†^	69.3^†c^	[11]
Ecallantide	7.1	9.18	26.4	#
Phospholipase A_2_ (*N. sumatrana*)	16	1.38^†^	49.0^†c^	[11]
Filgrastim	19	2.52^a^	10.5^a^	#
rhGH	20	9.14	4.1	[23]
rCD4	50	3.42	4.9	[23]
rt-PA	63	37.2	8.1	[23]
Albumin	67	0.012^b^	7.7	+, [24]
CD4-IgG	98	0.157	6.3	[23]
Adalimumab	144	0.012	5.4	+
Eculizumab	148	0.022	7.7	#
Infliximab	149	0.016*	5.8*	[25,26,27]

Abbreviations: CL = clearance; V = volume of distribution; rhGH = recombinant human growth hormone; rCD4 = recombinant CD4; rt-PA = recombinant tissue plasma activator; CD4-IgG = CD4-immunoglobin. ^a^ Human weight is assumed to be 70 kg; ^b^ CL calculated from half-life and V; ^c^ V at steady state calculated from reported V of central compartment and V of peripheral compartment; * Weighted average; # Information from DrugBank (https://www.drugbank.ca/); + Information from eMC (https://www.medicines.org.uk/emc/) ^†^ Allometrically scaled CL and V were calculated using the following equation (based on human weight of 70 kg): $CL_{\left(human\right)}=CL_{\left(animal\right)}\times {\left(\frac{WT_{human}}{WT_{animal}}\right)}^{0.75}$, and $V_{\left(human\right)}=V_{\left(animal\right)}\times {\left(\frac{WT_{human}}{WT_{animal}}\right)}^{1}$.

## Appendix B

The prior parameter values of clearance, *CL* and volume of distribution, *V* were calculated by imputing the molecular weight (*MW*) of toxins in units of kDa into the regression equations from Figure A1 and Figure A2, shown below:

 lnCL=−0.04548×MW+2.566lnV=−0.01073×MW+3.117

Refer to Table A2 for other parameter values used in the simulation.

**Table A2:** Parameter values for the simulation.

Parameters	Parameter Values
$\sigma_{prior,lnCL}$	1.6528
$\sigma_{prior,lnV}$	0.7525
$\omega_{CL}^{2}$	0.175
$\omega_{V}^{2}$	0.0852
$\sigma_{prop}$	0.217
$\sigma_{add}$	0.001

Abbreviations: $\sigma_{prior,lnCL}$, prior uncertainty associated with the regression model for clearance; $\sigma_{prior,lnV}$, prior uncertainty associated with the regression model for volume of distribution; $\omega_{CL}^{2}$, between subject variance of clearance; $\omega_{V}^{2}$, between subject variance of volume of distribution, $\sigma_{prop}$, proportional error; $\sigma_{add}$, additive error.
