# Supplementary material for: The Influence of the Different Disposition Characteristics of Snake Toxins on the Pharmacokinetics of Snake Venom
Source: Toxins (Basel). 2020 Mar 16;12(3):188. doi: 10.3390/toxins12030188 (PMC7150903; doi:10.3390/toxins12030188)
Supplement: Supplementary file 1 [file toxins-12-00188-s001.pdf]

Toxins **2019**, *12*, 188; doi:10.3390/toxins12030188

Supplementary Materials: The Influence of the Different Disposition Characteristics of Snake Toxins on the Pharmacokinetics of Snake Venom

Suchaya Sanhajariya, Geoffrey K. Isbister and Stephen B. Duffull

S1. The "Perfect Case" Scenario

The venom data generated were modelled under an intravenous bolus one-compartment model for each toxin using the population parameter estimates without uncertainty in the prior, i.e. setting the variances of the prior σ to zero [perfect case].

Results

One hundred datasets, with each data set comprising 100 virtual patients and each providing 12 blood samples were evaluated. A summary of the preferred models is provided in Figure S1. In no circumstance was more than a five-compartment model preferred and hence data for the six- to nine- compartment models are not shown. The number of successful runs varied from 34 to 100%. In general runs other than Mixtures for C (mixtures of two toxins with similar molecular weights) provided good convergence properties.

The concentration-time data of mixtures containing two toxins (A1-3, B1-3, and C1-3) were best described by two-compartment model. Mixtures containing three or more toxins (D-J) were best described by three-compartment models, although some datasets preferred four-compartment models.

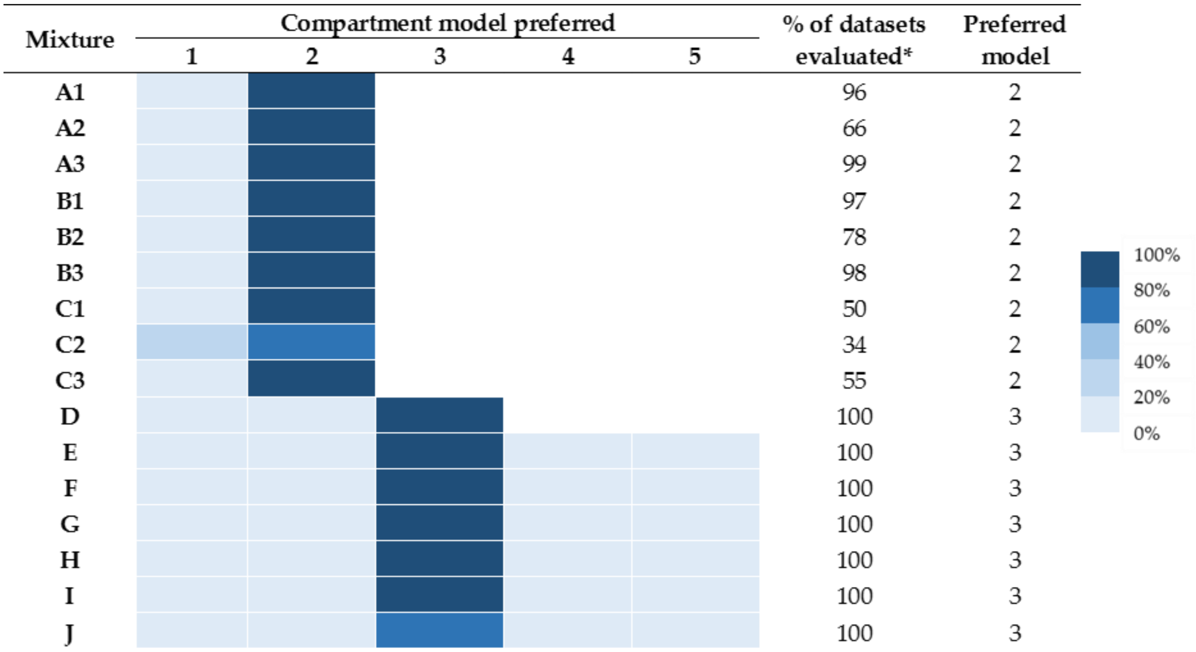

Figure S1.1. Fitting results of models under the "perfect case" scenario. Coloured chart represent the percentage of times the particular compartmental model was preferred. (*) denotes the % of successful runs.

S2. The Relationship between Molecular Weight of Toxins and Pharmacokinetic Parameter Values

In this *in silico* study, the pharmacokinetic parameters of toxins were simulated based on the relationship between molecular weight (MW) and pharmacokinetics (PK) parameter values of clearance and volume of distribution of protein based toxins. This supplementary material provides further examples that highlight this association and supports the use of MW as a predictor of the pharmacokinetics of protein/peptide based toxins in the body following envenomation.

Data for the first example is obtained from Sanhajariya et al. [1]. The PK parameters of various snake toxins following IV injection in rabbits and their corresponding MW are shown (Table S2.1). Note here that we only compare the PK parameters arise from studies a single species (in this case rabbits) for consistency in species. Figure S2.1 illustrates a simple log-linear relationship between MW and clearance and volume of distribution of toxins.

Table S2.1. PK parameters of toxins following IV injection in rabbits from Sanhajariya et al. 2018 [1].

Toxin	Snake	MW (kDa)	CL ($L \cdot h^{-1} \cdot kg^{-1}$)	V_{ss} ($L \cdot kg^{-1}$)
Neurotoxin	*N. sumatrana*	6.5	0.082	0.95[a]
Cytotoxin	*N. atra*	6.8	0.185	1.7[b]
Cardiotoxin	*N. sumatrana*	7	0.087	1.05[a]
PLA_2	*N. sumatrana*	16	0.048	0.7[a]
Habutobin	*T. flavoviridis*	28	0.061	0.05[a]

Abbreviations: MW, molecular weight; CL, systemic clearance; V_{ss}, volume of distribution at steady state; PLA_2, phospholipase A_2. [a] V_{ss} calculated from reported volume of distribution of central and peripheral compartment; [b] Reported as volume of distribution.

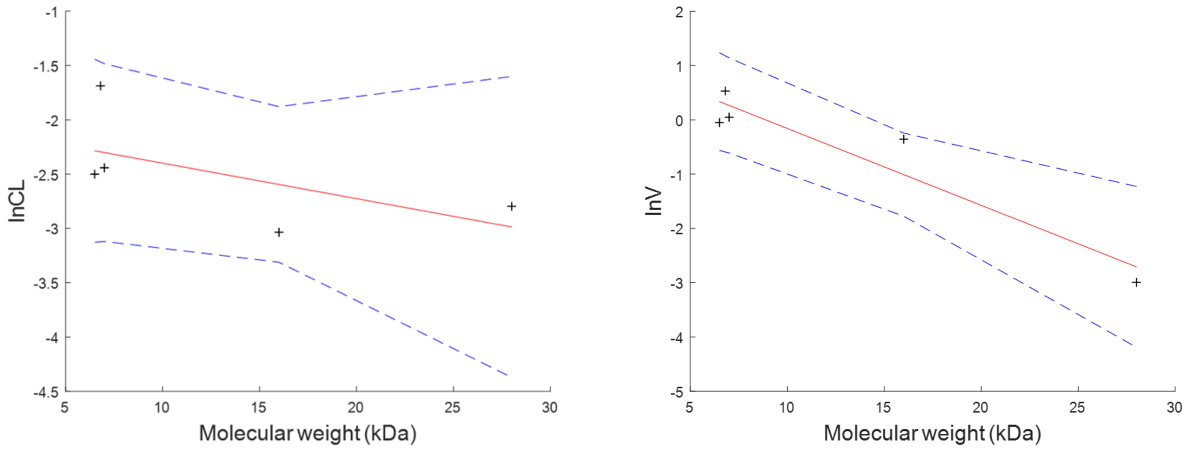

Figure S2.1. Plot of clearance (left) and volume of distribution (right) versus molecular weight of toxins when injected into rabbits. Red line is the least squares regression line and blue dash lines are 95% confidence interval. Regression equation: $lnCL = -0.0326 \times MW - 2.073$, uncertainty ($\sigma_{prior}$): 0.4713, $R^2 = 0.359$.
$lnV = -0.1416 \times MW + 1.256$, uncertainty ($\sigma_{prior}$): 0.5038, $R^2 = 0.9023$.

For the second example, the PK data of various therapeutic peptides with different MWs in human were extracted from Diao et al. [2] (shown in Table S2.2). The relationship between MW and PK parameters are shown in Figure S2.2.

Table S2.2. PK parameters of therapeutic peptides from Diao et al. [2].

Molecule	Route	MW (kDa)	CL($L \cdot h^{-1}$)	V_{ss} (L)	Half-life (h)
Exenatide	*SC*	0.4	8.2	28.3[a]	2.4
Ipamorelin	*IV*	0.7	5.4	15.5	2
Desmopressin	*IV, IM, SC, nasal, oral*	1.07	9.3	26.8	2
Leuprorelin	*IM depot, IV*	1.2	7.4	32[b]	3
Cetrorelix	*SC*	1.43	3.9	70	12.3
Degarelix	*SC*	1.69	0.03	55.7	1272
Liraglutide	*SC*	3.75	0.6	11.2[a]	13
Peginesatide	*SC, IV*	45	0.04	3.5[a]	55.3
Romiplostim	*SC*	60	0.02	4.8[c]	120–160

Abbreviations: MW, molecular weight; CL, systemic clearance; V_{ss}, volume of distribution at steady state. CL values were calculated from reported half-life and volume of distribution. [a] Reported as volume of distribution; [b] Average of the reported volume of distributions; [c] Reported as volume of distribution of central compartment.

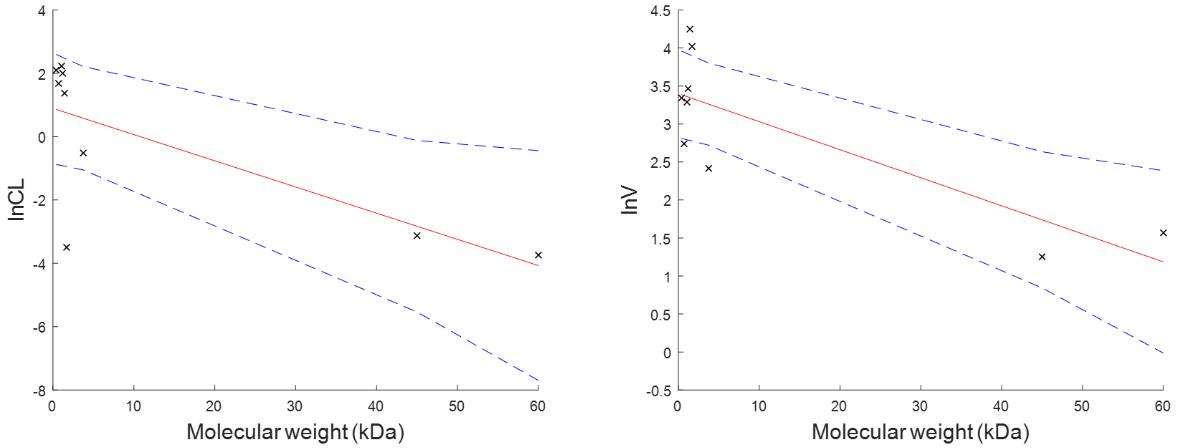

Figure S2.2. Plot of clearance (left) and volume of distribution (right) versus molecular weight of therapeutic peptides. Red line is the least squares regression line and blue dash lines are 95% confidence interval.
Regression equation: $lnCL = -0.0827 \times MW + 0.893$, uncertainty ($\sigma_{\text{prior}}$): 1.9104, $R^2 = 0.5276$.

$lnV = -0.0369 \times MW + 3.4$, uncertainty ($\sigma_{\text{prior}}$): 0.6321, $R^2 = 0.6702$.

Figures S2.3 and S2.4 compare the relationship between PK parameters (clearance, S2.3 and volume of distribution, S2.4) from above studies [1,2] (rabbits data were allometrically scaled to human values) with our current manuscript. The PK parameter values and MW of different proteins display a negative correlation in above studies [1,2] which is similar in correlation and relationship to those reported in our current manuscript. This highlights the association between the PK of proteins and their corresponding MW, and that MW is an important predictor of the values of clearance and volume of distribution of the toxins. Hence, this relationship may be used as a basis to help predict the PK profile of each toxin based on their MW.

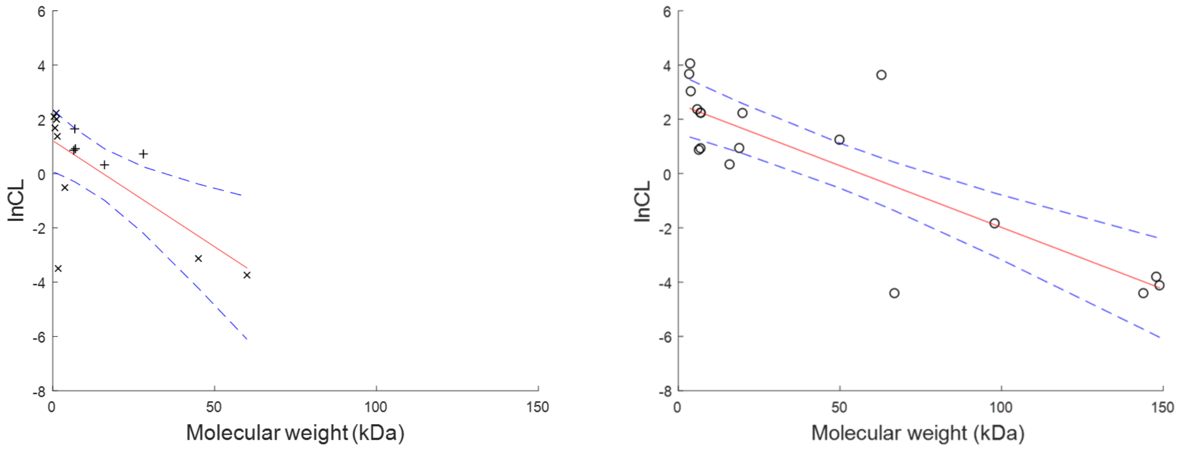

Figure S2.3. [LEFT]: Plot of clearance versus molecular weight of toxins when injected into rabbits (allometrically scaled to human values) from Sanhajariya et al. 2018 [1] (+) and therapeutic peptides from Diao et al. [2] (×). Regression equation: $lnCL = -0.0782 \times MW + 1.215$, uncertainty ($\sigma_{\text{prior}}$): 1.6062, R^2 = 0.4719. [RIGHT]: Plot from the manuscript. Regression equation: $lnCL = -0.04548 \times MW + 2.566$, uncertainty (σ_{prior}): 1.6528, R^2 = 0.7005. Red line is the least squares regression line and blue dash lines are 95% confidence interval.

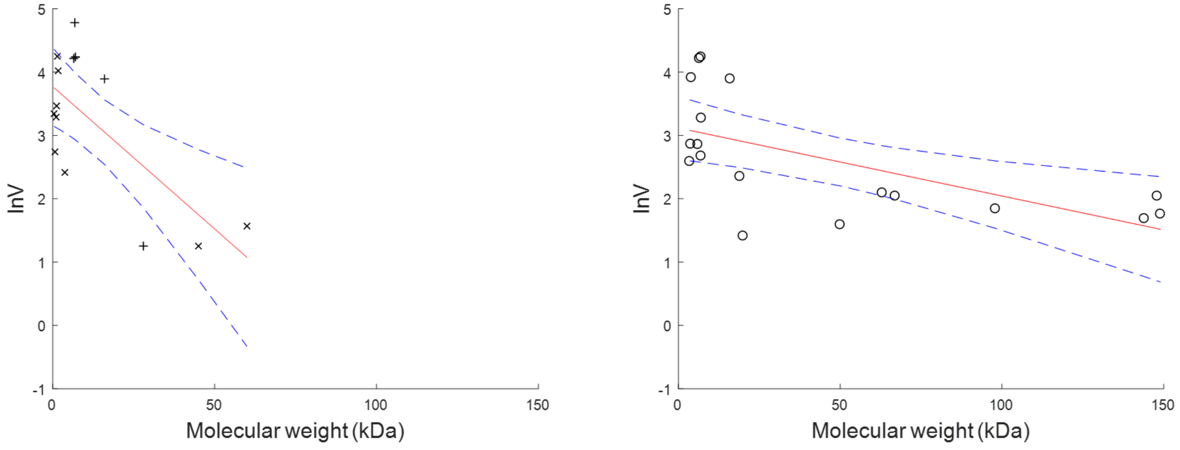

Figure S2.4. [LEFT]: Plot of volume of distribution versus molecular weight of toxins when injected into rabbits (allometrically scaled to human values) from Sanhajariya et al. 2018 [1] (+) and therapeutic peptides from referenced Diao et al. [2] (×). Regression equation: $lnV = -0.0449 \times MW + 3.771$, uncertainty ($\sigma_{\text{prior}}$): 0.8605, R^2 = 0.5069. [RIGHT]: Plot from the manuscript. Regression equation: $lnV = -0.01073 \times MW + 3.117$, uncertainty ($\sigma_{\text{prior}}$): 0.7525, R^2 = 0.386. Red line is the least squares regression line and blue dash lines are 95% confidence interval.

References

1. Sanhajariya, S.; Duffull, S.B.; Isbister, G.K. Pharmacokinetics of snake venom. *Toxins* **2018**, *10*, 73.
2. Diao, L.; Meibohm, B. Pharmacokinetics and pharmacokinetic–pharmacodynamic correlations of therapeutic peptides. *Clin. Pharmacokinet.* **2013**, *52*, 855–868.
